# Editorial: Reviews in animal welfare

**DOI:** 10.3389/fvets.2024.1485518

**Published:** 2024-09-23

**Authors:** Edward Narayan, Barbara Padalino

**Affiliations:** ^1^School of Agriculture and Food Sustainability, Faculty of Science, The University of Queensland, Brisbane, QLD, Australia; ^2^Centre for Animal Science, Queensland Alliance for Agriculture and Food Innovation, The University of Queensland, Brisbane, QLD, Australia; ^3^Division of Animal Sciences, Department of Agricultural and Food Sciences, University of Bologna, Bologna, Italy; ^4^Faculty of Science and Engineering, Southern Cross University, Coffs Harbour, NSW, Australia

**Keywords:** animal welfare, farming and wildlife, health, food, Five Domains Model

During the past decade, the term “welfare” has been widely discussed in animal industries. The growing number of wildlife-human conflicts caused by urban expansion and climate change reveals the urgent need of solutions that can ensure both wildlife welfare and human development requirements. In terms of farm animals, with the promotion of intensive production, whether intensification is leading to the suffering and poor welfare of animals has been a highly debated Research Topic. Pet species such as cats and dogs also received widespread welfare concerns to improve their quality of life. However, some issues related to animal welfare have been studied less in depth than others, more research is warranted to understand animal welfare issues across animal production systems and to come up with innovative solutions to improve animal health and wellbeing.

In this edition, we focussed on discussing our current knowledge of animal welfare. This Research Topic invited reviews to demonstrate available technologies, assessment tools, and methodologies that can be used to quantify and monitor animal welfare. Papers also explored the quality and validity of animal welfare related scientific research design and writing. Emerging Research Topics such as positive welfare was also covered in this Research Topic.

We collected 10 reviews (and one research article) on animal welfare Research Topics, related to a variety of species, from companion animals to livestock, and from laboratory animals to wild animals. The papers identified current gaps of knowledge and recent advancements in the field of animal welfare.

The first manuscript by Adamaκopoulou et al. demonstrated how “cats and dogs” related welfare literature has evolved worldwide over the last 40 years. The authors described the main research interests and discussed gaps in knowledge by conducting a search using Scopus^®^ and applying an innovative text mining approach. The study included a total of 1,775 scientific literature records that probed into cats' and dogs' welfare aspects. Descriptive statistical results, which were based on titles and abstracts of the records showed an increasing number of studies on this Research Topic while researchers in Europe and North America showed strong interest in cat and dog welfare research. Keywords such as “behavior,” “owner,” and “adopt” were frequently mentioned with “shelter” being the most used word in literature records. Some of the other keywords identified in this review included “stress and housing conditions,” “welfare and pain assessment,” “shelter management,” “euthanasia,” and “owners” and veterinarians' perceptions' of cats and dogs welfare.

In the second Research Topic, Masebo et al. analyzed the welfare of camels. Specifically, the authors studied intensive management system and regulations by reviewing 43 years of main Research Topics on camel welfare. The researchers suggested that over this period, attention has been raised to camel welfare with Asian authors contributing greatly to academic research. Among the 234 literature records analyzed in this study, “milk,” “calve,” “behavior,” “female,” “breed,” and “stable” were notable keywords. Beginning with the oldest Research Topics such as “female and male reproduction,” researchers over time covered a wide range of Research Topics like calf management, milk production, health and management system, behavior, and feeding.

The third paper by Littlewood et al. provided introduction to the concept of agency, and discussed the relationship between agency and positive emotions, experience, and welfare. The discussions were based on The Five Domains Model, which was updated in 2020 and redefined the Domain 4 from “Behavior” to “Behavioral Interactions.” The authors illustrated how the renamed domain can be used to evaluate positive animal welfare and took captive sugar gliders and racing greyhounds as real-life examples to demonstrate the applications of this domain.

The fourth paper was from the researchers Yan et al. who conducted qualitative evaluation of laboratory animal welfare among a total of 150 undergraduate and 148 postgraduate veterinary medicine students in China. The results given by respondents showed an overall strong sense of responsibility among students who were engaged in animal experiments, laboratory teaching, and learning to support and contribute to the improvement of laboratory animal welfare. However, the authors also pointed out that despite the passion, basic theoretical knowledge of animal ethics, adequate compass of experimental techniques and awareness of the existence of related supervisory agencies were still insufficient among current students. This study aimed to lay the cornerstone for the future of veterinary education and the development of humanity, compassion, as well as professional skills of veterinary students.

The fifth study by Whitham and Miller reviewed the existing evidence of affective state related vocal production in non-human mammals, and current available non-invasive methods used to investigate vocal activities. The authors highlighted that apart from negative contexts measurements such as pain levels and social isolation, acoustic activity can also be utilized as an indicator of positive affective state. Such vocalization is produced when animals are foraging, playing, grooming, or interacting with their intimate fellows. Related to cardiac activity, respiration rates, and hypothalamus-pituitary adrenal (HPA) axis activity, vocalization can be an effective tool for wildlife scientists to identify welfare-associated events. Vocalization examination discussed in this study could be achieved by applying modern acoustic monitoring systems and were valuable for practical situations such as husbandry routine or environment management, animal transfers, and introductions to monitor animal welfare and quality of life.

The sixth manuscript by Ghimire et al. focused on a controversial Research Topic- the welfare of the Asian elephants *(Elephas maximus)* that were involved in religious activities and in the logging or tourist industries. In this paper, the authors reviewed a variety of available animal welfare assessment tools (e.g., ZooMonitor, WelfareTrack, ZIMS, etc.), with a special emphasis on elephants (Elephant Behavioral Welfare Assessment Tool, Elephant Welfare Initiative, etc.). The toolkit includes methodologies based on various resources such as digital information systems, paper-based work, keeper ratings, welfare grading scales, etc. However, considering the multiplex captive environments of elephants throughout Asia, the authors also pointed out that further development of a comprehensive and practical tool is necessary.

The seventh paper by Sundman et al. investigated the welfare issue related to ill and injured feedlot cattle. In this study, the authors reviewed a total of 110 articles, in which only 12 articles mentioned the management of ill and injured cattle in specialized hospitals and two discussed the application of chronic pens. Although diagnoses such as Bovine Respiratory Disease Complex (BRDC), lameness, and gastrointestinal problems are very common in feedlots, these results indicated the current knowledge gaps in the negative valence of animal welfare caused by illness and injury in feedlot cattle. Sundman et al. also explored the potential of the Five Domains Model in the individual management of sick cattle, aiming at strengthening the welfare assurance in current industry practices and assisting producers toward understanding their animals' behavioral needs.

In the eighth study, Neves et al. probed into the causes of unsatisfying reproducibility in scientific research that require animal experiments. The authors noted that their review of 124 journal articles suggests that low quality and transparency of scientific writing, such as deficiency in data specification and description of crucial details, are the main causes of irreproducibility. The articles evaluated showed an assured demand for practical application of the 3R's principle (Refine, Reduce, and Replace) and international guidelines in experiment design, especially in areas such as metabolism, immunity, hormones, and stress to further improve the overall welfare and reduce the number of laboratory animals required in experimental protocols, avoid unnecessary financial input, and at the same time, alleviate compassion fatigue found in veterinarians and laboratory animal technicians.

The ninth review by Linstädt et al. covered a total of 2,818 English and German publications from 2011 to 2021 in five databases. This study systematically discussed the reliability of various welfare evaluation methods that can be applied in different environments such as farming systems and pasture-based systems. The authors summarized the validity of stress monitoring parameters such as hair cortisol concentration, heart rate variability, as well as biomarker research and behavioral studies. The authors also discussed emerging tools such as Precision Livestock Farming and Animal Need Index and Herd Data, aiming at improving the efficiency of livestock managers in evaluating animal welfare and identifying potential welfare concerns. At the end of this paper, Linstädt et al. recommended that importance should be attached to easily observable indicators such as lameness and body condition score in welfare assessment.

In the 10th study, Rokade et al. investigated the attitude of egg producers in India toward the global shift from the traditional battery cage poultry production system to the cage-free egg production system. While deficiencies such as limited space for movement in the cage, and inappropriate flooring can commonly be found in battery cage systems, cage-free systems are now regarded as a less cruel type of system, providing better welfare to the hens. This needs-assessment survey also highlighted producers' requests for corresponding support like financial assistance and technical training from both government and privates, to better cope with the building and development of the cage-free sector and be able to compete with battery cage poultry producers in the market.

In the 11th and final Research Topic, Fox et al. used 70 topknot wool samples in non-invasive cortisol and testosterone assessment to quantify the stress levels that rams were experiencing in Queensland, Australia. The authors also analyzed the potential relationship between the two different hormones. As an important indicator of stress response in animals, cortisol levels studied in this research provided important information on the quality of husbandry management and explore the large margin to improve animal welfare in the sustainable sheep industry. In recent years, non-invasive hormone assessment has been utilized by producers and researchers as an effective tool to assess long-term, historic reflections of stress levels, without being affected by acute stressors as sampling at the time of collection.

Overall, this Research Topic highlights some of the recent developments in the broad field of animal welfare assessment in different species.

